# Influences of thermal environment on fish growth

**DOI:** 10.1002/ece3.3239

**Published:** 2017-07-26

**Authors:** Sebastián Boltaña, Nataly Sanhueza, Andrea Aguilar, Cristian Gallardo‐Escarate, Gabriel Arriagada, Juan Antonio Valdes, Doris Soto, Renato A. Quiñones

**Affiliations:** ^1^ Interdisciplinary Center for Aquaculture Research (INCAR) Department of Oceanography Biotechnology Center University of Concepción Concepción Chile; ^2^ Facultad de Ciencias Biológicas Universidad Andrés Bello Santiago Chile

**Keywords:** Atlantic salmon (*Salmo salar*), ectotherm, growth trajectory, metabolism, muscle cellularity, physiology

## Abstract

Thermoregulation in ectothermic animals is influenced by the ability to effectively respond to thermal variations. While it is known that ectotherms are affected by thermal changes, it remains unknown whether physiological and/or metabolic traits are impacted by modifications to the thermal environment. Our research provides key evidence that fish ectotherms are highly influenced by thermal variability during development, which leads to important modifications at several metabolic levels (e.g., growth trajectories, microstructural alterations, muscle injuries, and molecular mechanisms). In Atlantic salmon (*Salmo salar*), a wide thermal range (Δ_T_ 6.4°C) during development (posthatch larvae to juveniles) was associated with increases in key thermal performance measures for survival and growth trajectory. Other metabolic traits were also significantly influenced, such as size, muscle cellularity, and molecular growth regulators possibly affected by adaptive processes. In contrast, a restricted thermal range (Δ_T_ 1.4°C) was detrimental to growth, survival, and cellular microstructure as muscle growth could not keep pace with increased metabolic demands. These findings provide a possible basic explanation for the effects of thermal environment during growth. In conclusion, our results highlight the key role of thermal range amplitude on survival and on interactions with major metabolism‐regulating processes that have positive adaptive effects for organisms.

## 1 INTRODUCTION

Temperature variations diversely affect fish species, and while information is growing regarding the effects of thermal variation on aquatic systems, limited knowledge is available on impacts to the metabolic and physiological traits of fish. Thermal change is the strongest influencing force for ectothermic organisms such as fish, which, in contrast to endotherms, cannot maintain a constant body temperature via homeostatic mechanisms (Angilletta, 2009; Heinrich, 1993; Mednikov, 1977; Stevenson, 1985). In aquatic systems, fish are exposed to spatial and temporal variations in temperature that significantly affect individual physiological traits (i.e., growth and metabolic condition), genetic structure (Bradbury et al., 2010), and/or survival (Houde, 2008). Indeed, species‐specific thermal tolerances are a primary driver establishing the environments in which fish live (Bogert & Cowles, 1944; Golovanov, 2006; Kearney, Shine, & Porter, 2009; Killen, 2014; Reynolds, 1978).

Through thermoregulation, organisms can integrate temperature with physiological demands, resulting in an attraction to suitable warmer or colder environments (Boltaña et al., 2013; Burton, Killen, Armstrong, & Metcalfe, 2011; Killen, 2014; Killen, Marras, Metcalfe, McKenzie, & Domenici, 2013; Rey, Moiche, Boltaña, Teles, & MacKenzie, 2017). Thermoregulation can imply changes in individual physiological traits, including, among others, standard metabolic rate (Burton et al., 2011) or absolute aerobic scope (Norin & Malte, 2011; Pörtner & Farrell, 2008), which deeply affect individual thermal adaptations (Khan & Herbert, 2012; Munoz, Farrell, Heath, & Neff, 2015; Norin, Malte, & Clark, 2014). Changes in the metabolic machinery (e.g., resulting in increased muscle fiber) are intrinsically coupled with and crucial for the dynamic processes of thermoregulation (Killen, Atkinson, & Glazier, 2010). Importantly, metabolism is also directly influenced by thermal variations. For example, a restricted thermal range not only modifies organism behaviors, but can also determine the occurrence of metabolic changes and acceptable metabolic or physiological modifications (Dell, Pawar, & Savage, 2011; Farrell et al., 2008). Therefore, thermoregulation systemically implies fine‐tuned mechanisms to modulate specific temperature effects in biological processes.

Variances in thermal range may also result in changes to growth trajectories (Fangue, Podrabsky, Crawshaw, & Schulte, 2009; Killen, 2014) and, by extension, in muscle structure (Matteini et al., 2012; Bryan, Kelsch, & Neill, 1990). Research data suggest that contrasting temperatures trigger temporal and spatial structural changes to the collagen denaturation of muscle fibers and myofibrils (i.e., muscle structure) (Brüggemann, Brewer, Risbo, & Bagatolli, 2010; Campos et al., 2013; Matteini et al., 2012). Therefore, individuals reared in a limited thermal range are expected to have increased maintenance costs, as reflected by muscle myopathies and low growth trajectories (Bryan et al., 1990; Priede, 1985). Second‐harmonic generation (SHG) microscopy can be used to inspect fibrillar collagen degradation (Huang et al., 2011) and to examine whether sarcomere structure is influenced by thermal range amplitude. In turn, to deduce if thermoregulatory range drives dissimilar metabolic traits, cellular muscle analysis is an option for unraveling divergent muscle‐growth patterns, namely of growth contributions via hyperplasia (i.e., increased fiber quantity) and hypertrophy (i.e., increased fiber diameter) (Johnston, 2006; Johnston et al., 2009; Zhu et al., 2014).

Atlantic salmon (*Salmo salar*), an anadromous fish species, live in streams, rivers, and open‐ocean marine environments, thus experiencing spatial and temporal temperature fluctuations that influence survival and growth (Crozier, Zabel, & Hamlet, 2008). *S. salar* is also one of the most successfully farmed species in aquaculture, and farming practices have somewhat adapted to the anadromous behavior of *S. salar* by mimicking the different environments over the distinct developmental stages (Torrissen et al., 2011). Nevertheless, farmed fish are more‐or‐less restricted to spatially constant temperature conditions within the containment unit. This contrasts with wild fish, which are freely mobile and can choose different conditions according to physiological needs, the search for food, to escape predators, and other factors.

In accordance with the above, the aim of this study was to investigate the degree of influence that spatial variation in temperature range has on *S. salar* survival, muscle structure, growth trajectory, and the genic expression of mRNAs tightly linked to hyperplasic/hypertrophic growth. We hypothesized that fish responses to spatially variable temperatures would involve temperature‐dependent effects related to metabolic demands, such as growth, and survival during development. Understanding the effects of the interaction between thermal environment and regulatory processes is relevant for identifying mechanisms impacted by environmental fluctuations under both fish‐farming and wild conditions, such as those driven by climate change. Therefore, the following questions were addressed:

1. What is the role of spatial thermoregulatory range on survival during *S. salar* development?
2. How does the amplitude of temperature range affect growth trajectories and muscle structure?
3. What changes occur in the regulatory mechanisms of muscle growth due to different thermal ranges?

## 2 MATERIALS AND METHODS

### 2.1 Fish husbandry and experimental conditions

All *S. salar* thermal experiments were carried out at the ThermoFish Lab, Biotechnology Center, Universidad de Concepción, Concepcion, Chile. The selected study species, *S. salar,* is a common, widespread anadromous Atlantic fish. Specimens were handled in accordance with the “International Guiding Principles for Biomedical Research Involving Animals” established by the European Union Council (2010/63/EU). *S. salar* embryos were obtained in December 2015 from AquaGen S.A. (Melipeuco, Chile). All embryos were from the same batch and, consequently, had equal genetic backgrounds.

Fish embryos were initially maintained in a temperature‐controlled room (18°C). Two recirculating freshwater systems (210 × 150 × 90 cm) were used, with each system using UV‐sterilized water and a flow rate of 5 m^3^ hr^−1^. Each system contained three independent tanks (60 × 140 × 70 cm). The water temperature of each tank was measured twice per day (7 ± 0.7°C). Dissolved oxygen was also measured daily and always remained above 9 mg/L^−1^. Ammonia, nitrite, and pH were measured twice per week. Total ammonia and nitrite concentrations in each tank were maintained under 0.05 and 0.01 mg L^−1^, and pH remained at 8.0 ± 0.5. A 24‐hr dark cycle photoperiod was used until the embryos hatched.

After 30 days, 95% of the embryos hatched. After the yolk was fully absorbed (40 days posthatching, [dph]), water temperature was increased by 1°C per hour until reaching the required thermoregulatory ranges. The larvae were gradually acclimatized to a 12‐hr light: 12‐hr dark photoperiod cycle. As a note, *S. salar* specimens were raised from first feeding to 10 months posthatching under a 12‐hr light: 12‐hr dark photoperiod to artificially reproduce the Autumn–Winter seasons, in correspondence with the annual cycle of species (Davidson et al., 2016). The larvae were fed a maintenance diet (Biomar, S.A., Puerto Montt, Chile) twice daily for 9 months.

**Figure 1:**
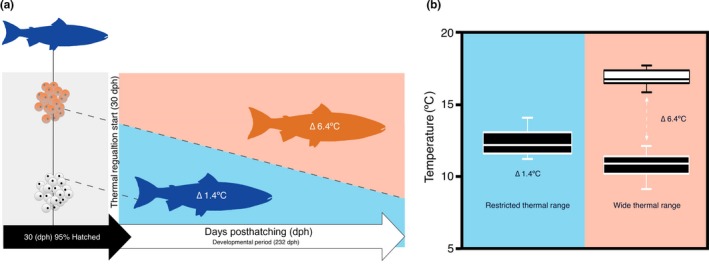
Experimental design and thermoregulatory limits. (a) Experimental design diagram showing both thermoregulatory treatments (i.e., restricted thermoregulatory range; Δ_T_ 1.4°C and wide thermoregulatory range; Δ_T_ 6.4°C) for *S. salar* rearing over a developmental period (232 dph). Temperature treatments are color‐coded, and the experimental duration for each developmental period is indicated in horizontal gray boxes. The vertical gray arrow indicates the start of the divergent thermoregulatory treatments. (b) The thermoregulatory ranges used during *S. salar* development are indicated, where blue represents the restricted (Δ_T_ 1.4°C) and red represents the wide range (Δ_T_ 6.4°C). The white and black boxplots show the upper and lower temperature limits, with the whiskers representing the range. The boxplots are positioned in the data midway point, and the horizontal line indicates the median. The delta (Δ_T_) values represent the thermoregulatory range of the thermal treatments

All experiments were performed in a temperature‐controlled room (12°C). Fish were randomly assigned to two thermal treatment groups according to Boltaña et al. (2013). Experiments were performed using an in‐house, custom‐built tank system with three replicate tanks per group. The temperature gradients of both groups were established using an external water jacket system set at different temperatures. This setup provided a continuous vertical thermal gradient within the tanks, thus generating two treatment conditions (Figure 1): (1) wide thermal range (WTR), Δ_T_ 6.4°C (*T*_min_ 9.8°C to *T*_max_ 16.4°C) from top to bottom, respectively, thereby mimicking a natural thermal gradient and (2) restricted thermal range (RTR), Δ_T_ 1.4°C (12.7 ± 1.4°C). Water temperature in the vertical column was recorded by thermal sensors located at different points within the water column (Thermocouple thermometer 53/54 II; Fluke^®^ Corporation, Washington, USA). No significant differences were recorded in oxygen levels throughout the gradient. In the WTR, the temperature gradient was constantly maintained (i.e., 24/7). The gradient ranges were set based on (1) the natural thermal range of *S. salar*, which is between 7 and 22°C (Torrissen et al., 2011) and (2) constant laboratory conditions that considered the most common land‐based farming conditions.

To answer the aforementioned research questions, samples were collected from each group at distinct time points for posterior morphometric/allometric analyses. Additionally, SHG microscopy was employed as a powerful technique for imaging fibrillar collagen in muscle. This assay was chosen to potentially demonstrate the sensitivity of the sarcomere structure to distinct thermoregulatory range amplitudes. Furthermore, correlations were performed to assess whether temperature preferences of individual fish drive unlike metabolic traits. This was achieved using a combination of analyses on muscle cellularity sections to unravel patterns of hyperplasic and hypertrophic growth. Finally, the expressions of muscle‐growth‐related genes were evaluated to establish if molecular regulation is influenced by thermal range.

### 2.2 Biological stages and morphometric analysis

Random samples (*n* ≥ 6 per group) were taken at the following development stages: (1) hatching, 0 dph; (2) onset of external feeding (15 dph); (3) mid‐larval stage (40, 55, and 78 dph); (4) advanced larval stage (92, 100, 120, 134, 148, and 162 dph); and (5) juvenile stage (192, 204, 118, and 232 dph). Sampled fish were anaesthetized with MS‐222 (3‐aminobenzoic acid ethyl ester; Sigma, Vienna, Austria), and body length and weight were measured. The fish were then fixed in absolute ethanol, and the epaxial muscle, located between the cephalic region and anal fin, was dissected and treated with 30% saccharose in demineralized water. Epaxial blocks were frozen in isopentane (2‐methyl butane) cooled to near freezing (−159°C) in liquid nitrogen. Frozen sections were cut on a cryostat at 10 μm transversal to the body axis at the dorsal fin level, mounted on slides coated with Vectabond (Vector Laboratories, Burlingame, CA, USA) to improve section adhesion and then stained with hematoxylin–eosin (Sigma).

Morphometric variables were also measured in transversal body sections of each fish at the dorsal fin level. Total cross‐sectional muscle area (A [mm^2^] [muscle]), total fiber number (*N* [fibers]) and fiber cross‐sectional area (F_A_ [μm^2^] [muscle fiber]) were measured. The total cross‐sectional muscle area (A [muscle]) and measured fiber quantity were computed after tracing the physical limits of interest in the section on the monitor (200× magnification). Fiber diameter (μm) was indirectly estimated as the diameter of a circle with the same fiber area position as a perfect cross section. This study was performed using an optical microscope and the Image‐Pro Plus 7 software (Media Cybernetics, Inc., Rockville, MD, USA). The relative contribution of hypertrophy and hyperplasia toward increases in the cross‐sectional area was estimated as reported elsewhere (Valente et al., 1999) and as shown in equation (1).

(1) $$\Delta A\left(\mu m^{2}\right)=N_{m}\Delta F_{A}\left(\mu m^{2}\right)+F_{A}\left(\mu m^{2}\right)\Delta N$$

where *Δ* is calculated between two consecutive sampling times (*t* and *t* + 1), and *N*_m_ and *F*_*A*_ refer to the mean total number of fast fibers and fiber area at *t*, respectively.

### 2.3 RNA extraction and cDNA synthesis

All samples were snap‐frozen in liquid nitrogen and conserved at −80°C until further analysis. Total RNA was extracted from epaxial muscle (100 mg) with the TRIzol Reagent (1 ml; Invitrogen, Carlsbad, CA, USA) and was quantified by absorbance at 260 nm. Only samples with an A260/280 ratio between 1.8 and 2.1, and an A260/230 ratio above 1.8 were used for reverse transcription. Purified RNA integrity was confirmed by agarose gel electrophoresis. cDNA was synthesized from total RNA (1000 ng μl^−1^) using the RevertAid H Minus First Strand cDNA Synthesis Kit (Fermentas, Waltham, MA, USA) according to the manufacturer's indications.

### 2.4 Absolute mRNA abundance quantification

qPCR analysis was performed using the Maxima SYBR Green qPCR Master Mix (2×) (Fermentas). cDNA used in qPCR assays was first diluted with nuclease‐free water (Qiagen). Each qPCR mixture contained the SYBR Green Master Mix, 2 μl cDNA, 500 nmol/L each primer, and RNase‐free water to a final volume of 10 μl. Amplification was performed in triplicate on 96‐well plates with the following thermal cycling conditions: initial activation for 10 min at 95°C, followed by 40 cycles of 15 s at 95°C, 30 s at 60°C, and 30 s at 72°C. A dilution series made from known concentrations of plasmid containing the PCR inserts was used to calculate absolute copy numbers for each of the genes examined. Previously published primers were used (Table S1).

### 2.5 Absolute quantification standards

An absolute quantification approach was used that involved calculating the number of gene copies in unknown “test” samples from comparison with a standard curve prepared using a dilution series of linearized plasmids with known concentrations (Pfaffl, 2004). The PCR product for each gene was extracted from agarose gel using the Nucleospin Gel and PCR Clean‐Up Kit (MACHEREY‐NAGEL, Dueren, Germany). The PCR amplicons were cloned using pGEM‐T Easy Vector and JM109 High‐Efficiency Competent Cells (Promega, Madison, WI, USA). The Nucleospin Plasmid Quick Pure Kit (MACHEREY‐NAGEL) was used to purify the plasmid DNA containing the PCR insert. Then, the plasmid was linearized using the HindIII restriction enzyme to prevent amplification efficiency problems that can arise from using supercoiled plasmids (Hou, Zhang, Miranda, & Lin, 2010), and the amount of dsDNA was quantified using the Quant‐iT PicoGreen dsDNA Assay Kit (Invitrogen). The concentration of each plasmid was calculated by absorbance at 260 nm, and a fivefold dilution series was produced for copy number calculations via qPCR and using equation (2).

(2) $$\text{Number}\;\text{of}\;\text{copies}=\frac{\text{amount}\;*\;6,022\times 10^{23}}{\text{length}*1\times 10^{9}*650},$$

where the *amount* of DNA (ng) was derived from absorbance at 260 nm and *length* (base pairs) was determined by adding the PCR product length to the size of the plasmid.

The use of these standard curves controlled for amplification efficiency differences between assays and permitted calculating the “absolute” number of mRNA transcripts, thereby facilitating gene comparisons.

### 2.6 Sarcomere length via second‐harmonic generation imaging

A pulsed near‐infrared laser (1064 nm, 7 ps, 76 MHz; PicoTran, High‐Q Laser, Rankweil, Austria) was used for excitation. The pulse train of the laser was directed to an inverted optical microscope (Eclipse Ti; Nikon, Tokyo, Japan) and focused onto the sample with a water immersion objective (CFI Plan Apo 60×, N.A. 1.2; Nikon). To eliminate the polarization dependence of the SHG signal, the laser light was converted to circular polarization before entering the microscope. The forward propagating signal was collected with a microscope condenser (N.A. 0.52), spectrally filtered with optical filters, and detected with a thermoelectrically cooled photomultiplier sensor (H‐7422‐40; Hamamatsu Photonics, Hamamatsu City, Japan). SHG signaling was confirmed through spectral analysis of the emission, which exhibited a single spectral line at 532 nm. To increase the signal‐to‐noise ratio, a lock‐in amplifier was employed; the SHG images were constructed via point‐by‐point recording of the demodulated signal while raster‐scanning the sample with respect to the fixed laser focus using a three‐axis scanning stage (P‐563.3CD; Physik Instruments, Karlsruhe, Germany), as previously described (Jhan et al., 2008; Wu et al., 2009). The laser power, measured at the focus, was typically between 40 and 60 mW. The temporal duration at each pixel was 10 ms. Sample scanning, signal collection, and image construction were completed with computer codes (LabView; National Instruments, Austin, TX, USA).

### 2.7 Statistical analyses

The overall influences of treatments and time on growth or muscle cellularity were modeled using ordinary least‐squares (OLS) regression models, with treatment, time, and the interactions thereof included as covariates. Fish length was log‐transformed to meet assumptions of normality and homoscedasticity. This particular analysis was performed with the Stata v14 program (StataCorp LP, College Station, TX, USA). All remaining statistical analyses were performed using the STATISTICA v8.0 software (StatSoft, Tulsa, OK, USA). Graphs were plotted with GraphPad PRISM v6.0 (GraphPad Software, Inc. California, USA) and MS Excel 2011. Gene expression data were further subjected to unsupervised hierarchical cluster analysis with the Babelomics 5 (http://www.babelomics.org/) using Pearson's correlation coefficient as a similarity measurement. For all tests, significance levels were set at *p *<* *.05. For SHG analysis, the means of the two groups were compared using a two‐tailed Student's *t* test, with statistical significance set at *p* < 10^−5^.

## 3 RESULTS

### 3.1 Growth and development under different thermal environments

**Figure 2:**
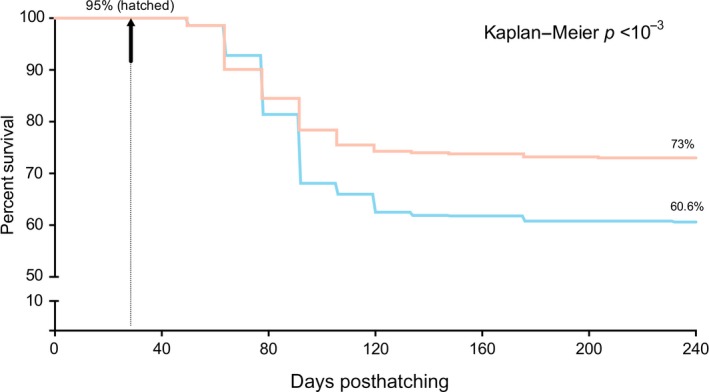
Survival percentage diagram. Kaplan–Meier analysis correlated the two thermoregulatory ranges (red = wide range and blue = restricted range) with the percent (%) of survival. The vertical arrow indicates the start of the thermoregulatory treatments

To test the presented research hypothesis, the effects of thermal range amplitude on *S. salar* survival during development were assessed. The experimental environment setup allowed fish to freely move throughout a temperature gradient (Δ_T_ 6.4°C) during the posthatch larvae to juvenile stages (232 dph). Another group of fish was kept within a restricted temperature range (Δ_T_ 1.4°C), that is, thermal distribution across the tank did not significantly vary (12 ± 1.4°C). *S. salar* larvae viability was determined in the two treatments (Figure 1). The tested thermic ranges had contrasting effects on the survival of *S. salar,* with the WTR yielding better survival performance than fish inhabiting the RTR (Figure 1, Figure S1). According to the Kaplan–Meier estimate for survival, these differences were significant after 90 dph and throughout the experimental period (Figure 2).

### 3.2 Impaired growth and muscular features

**Figure 3:**
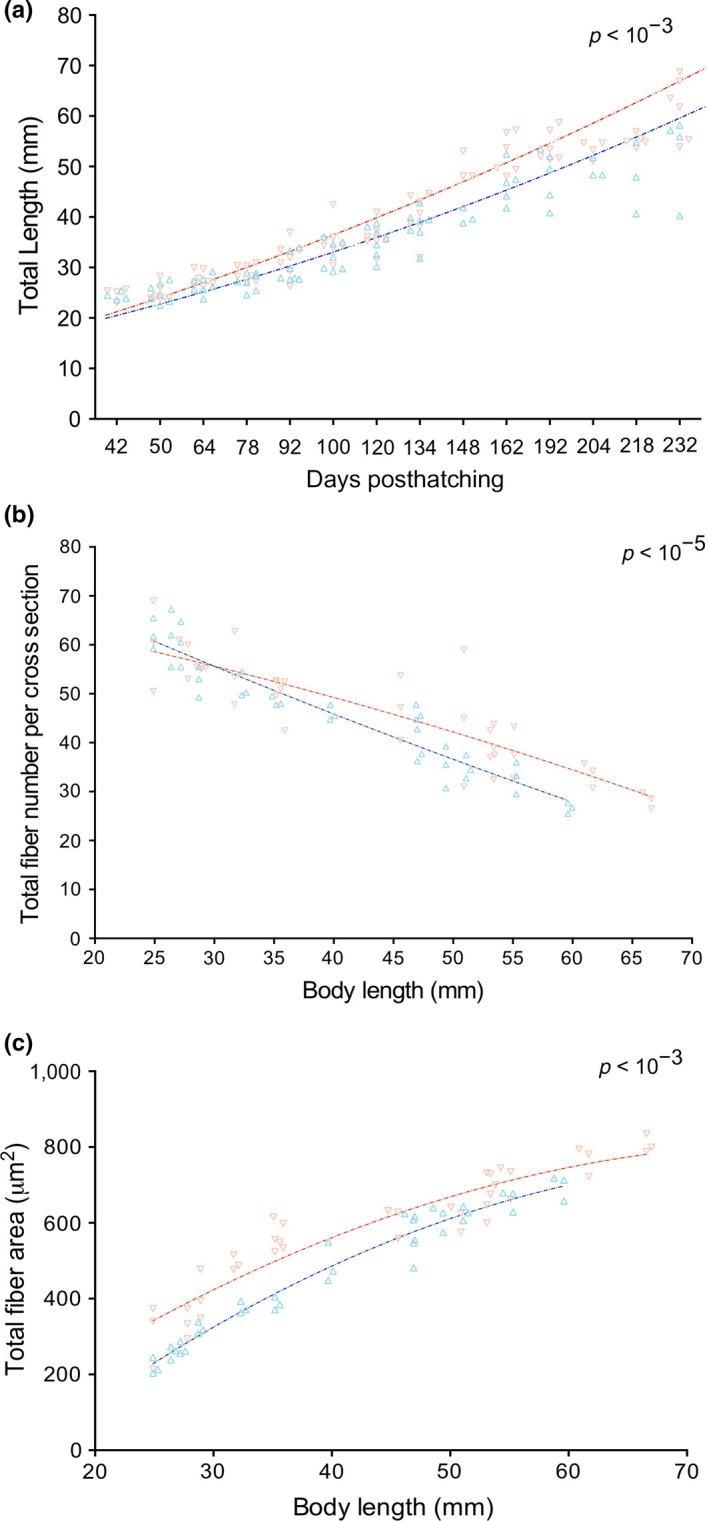
Growth measures after yolk sac absorption following 232 (dph) of thermal gradient treatments. (a) body length; (b) total fiber number *N,* (c) and total fiber area *A* (μm^2^) in fast muscle during fish development under different thermoregulatory ranges (red = wide range and blue = restricted range). Statistically significant differences were found between both thermal groups (*p* < 10^−3^)

While the mechanistic basis for thermoregulation is well‐known, current knowledge is limited to explaining the underlying effect of temperature range amplitude on metabolic traits or growth trajectories. Therefore, the present study reared *S. salar* larvae under wide and restricted thermal ranges and conducted morphometric analyses of both groups. Remarkable differences in individual morphometric parameters were found between the two treatments. The linear regression model (adjusted *R* ^2 ^= .906) indicated that WTR fish were larger (Figure 3a, red line), whereas RTR fish were smaller (Figure 3a, blue line, and Figure S2, Wald *p *=* *.021). RTR larvae also presented a significantly reduced quantity of muscle fibers as compared to WTR fish (Figure 3b; *F* test *p *< 10^−5^). Linear regression modeling (adjusted *R* ^2^ = 0.943) suggested that fiber area was significantly greater in WTR fish than RTR counterparts during most of the study period (Figure 3c, Figure S3; *F* test *p *< 10^−3^). Nevertheless, these differences were only significant at 110 and 128 dph (Wald *p *=* *.040 and *p *=* *.025, respectively). Based on the observed differences in growth trajectories, we postulate that a wide temperature range affects metabolic machinery, subsequently resulting in larger individuals than fish reared within a restricted temperature range.

**Figure 4:**
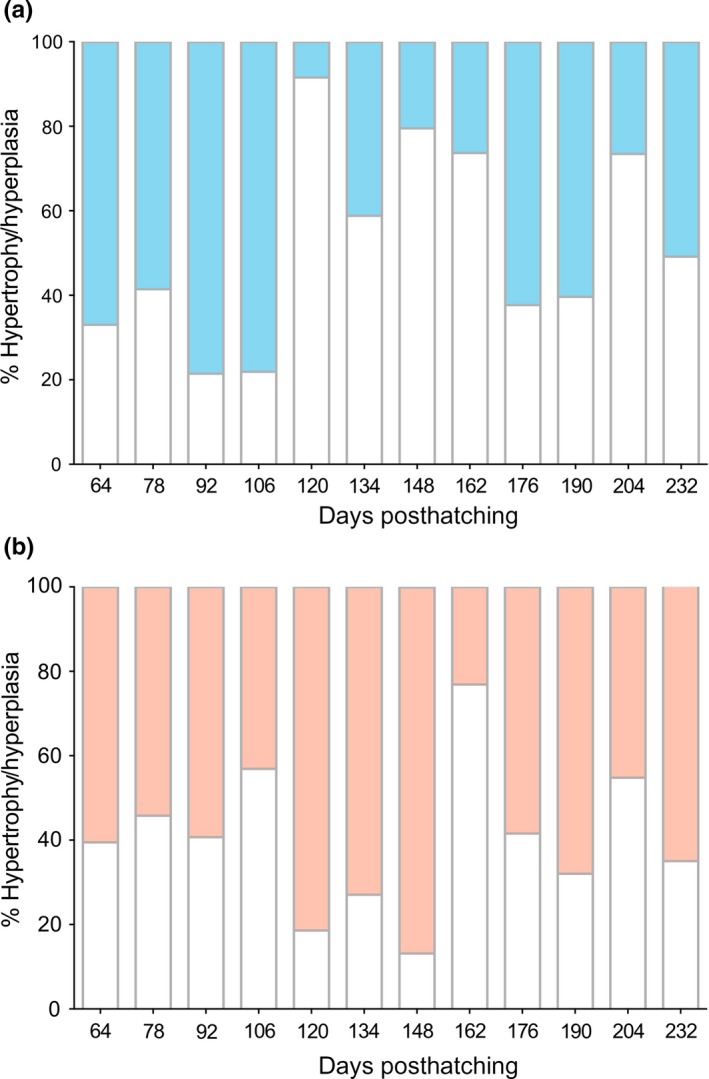
Contribution of hyperplasia and hypertrophy to white muscle growth. Shown are hyperplasic and hypertrophic muscle growths in fish reared under different thermal gradients during the development (232 days posthatching). (a) Restricted thermoregulatory range, blue. (b) Wide thermoregulatory range, red

To determine whether thermoregulatory behaviors simply induce shifts in the muscle cellularity profile by modifying relative contributions to hypertrophy/hyperplasia, muscle structures were contrasted to establish the influence of temperature range amplitude (Figure 4a and b). From hatching to the advanced larval stage, hyperplasic growth was the dominant process in both temperature ranges, as evidenced by an increased fiber quantity. From the advanced larvae to juvenile stages, hypertrophic growth became more predominant in the RTR group (71.3% ± 17.8% hypertrophy, Figure 4a). The relative contribution of hypertrophy and hyperplasia toward an increased cross‐sectional area of fiber diameters was right‐skewed for RTR fish, as evidenced by a higher percentage of thicker fibers (*F* test *p *<* *10^−3^, Figure 4a). In the WTR fish, hyperplasia contributed 60.6% ± 5.3% to increased muscle area from the larval (40 dph) to advanced larval (180 dph) stages, whereas hypertrophy accounted for 52.6% ± 9.6% of growth during the juvenile stage (Figure 4b). It is possible that the observed differences in muscle‐growth contributions were not a consequence of a temporal shift. Instead, the effects to muscle cellularity and fiber length might be linked to extended thermoregulation behaviors. Therefore, thermal range amplitude would, over time, result in an increased focalization of hypertrophy/hyperplasia mechanisms and functional specialization.

**Figure 5:**
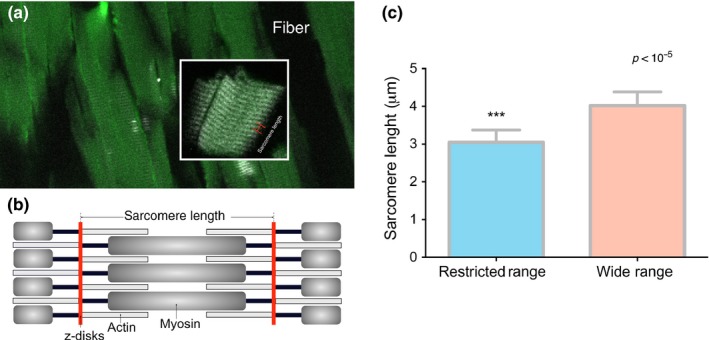
Sarcomere length unraveling detected by second‐harmonic generation imaging. (a) High‐resolution muscle image of *S. salar* sarcomeres. The inset shows the distinctive sarcomere myosin pattern (bar scale = 5 mm). (b) Diagram of sarcomere structure and key filament components. Sarcomere length is the distance between z‐disks. (c) Effect of thermoregulation range amplitude on sarcomere length. Blue restricted range individuals, and red bar wide range individuals (*n* = 24 for each thermoregulatory group). Values are shown as the mean ± SD. Differences were evaluated with a two‐tailed Student's *t* test (***p < 10^−5^)

To further highlight this effect, the structural components of the sarcomere muscle were analyzed. SHG analyses of RTR individuals revealed a consistently shorter sarcomere than WTR individuals_,_ although the morphological integrity of the myocyte in the RTR individuals remained intact (Figure 5c, 3.044 mm vs. 4.019 mm, *p *<* *10^−3^). This result suggests that the thermal environment influences sarcomere microstructure and, more specifically, that a RTR can induce a shorter sarcomere. The amplitude of the temperature range strongly affected muscle structure, with significant consequences for individuals reared in a RTR. The currently assessed restricted setup can be compared with the thermal environments often used in aquaculture or laboratory facilities, conditions under which fish evidence several responses that constrain the integration of regulatory systems.

### 3.3 Muscle‐growth gene expression analysis and thermoregulation range‐dependent mRNA abundances

**Figure 6:**
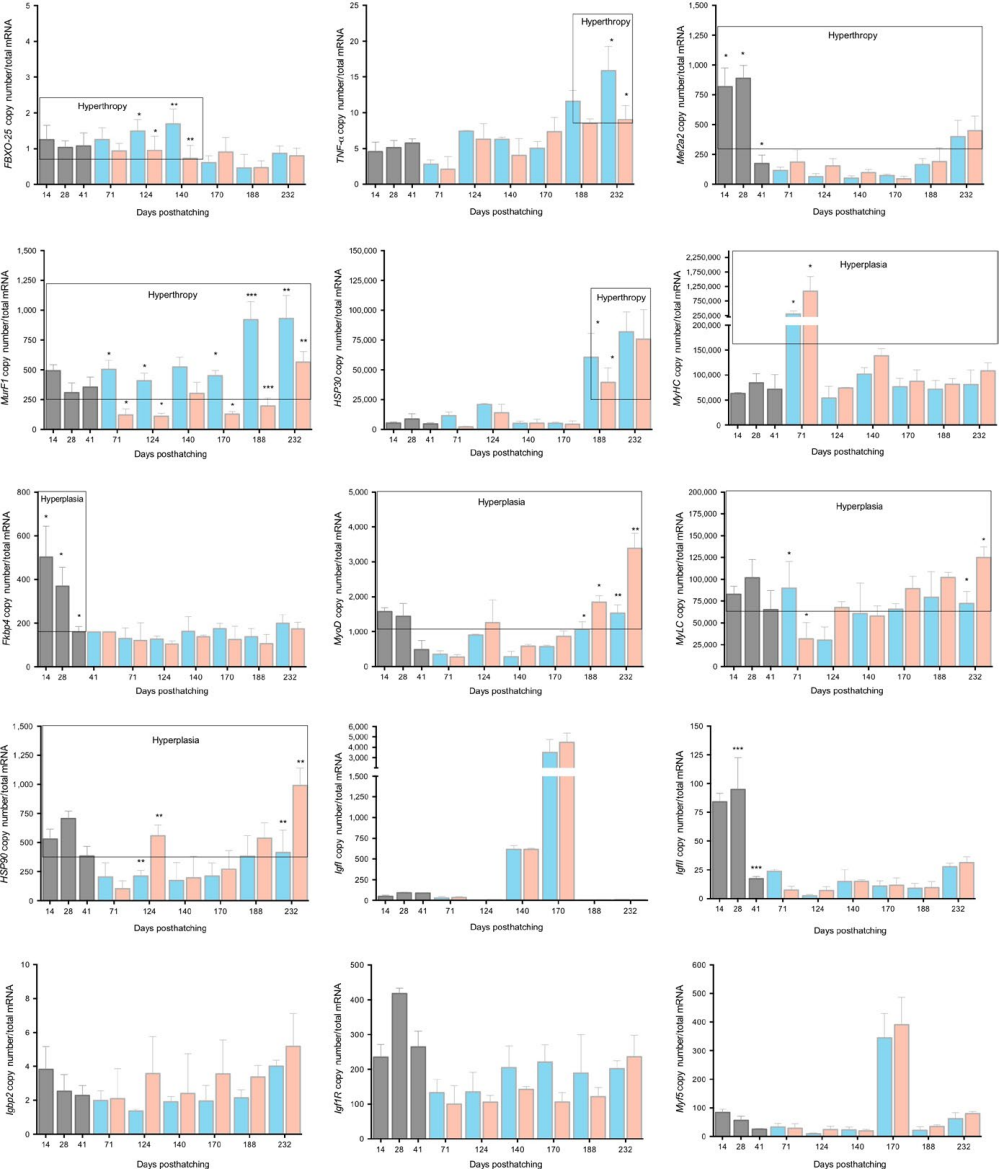
Expression profiles for 13 muscle‐growth pathway genes in developing *S. salar* muscle. Differences in mRNA abundances (back‐transformed, ordinary least‐squares means) between the thermal treatments (red = *T*_ch_, wide range and blue = *T*_r_, restricted range) are shown for each gene (*igf1, igf1r, igf2, hsp30, hsp90a1, myod, fbxo25, mef2a, Murf1, myhc, mlc2, fkbp4,* and *myf5*). Asterisks denote significantly different mRNA levels between the thermoregulatory ranges. Values are represented as the mean ± SD. Differences were evaluated with a one‐tailed ANOVA (*p < 10^−3^; ** *p < *10^−4^; *** *p < *10^−5^)

**Figure 7:**
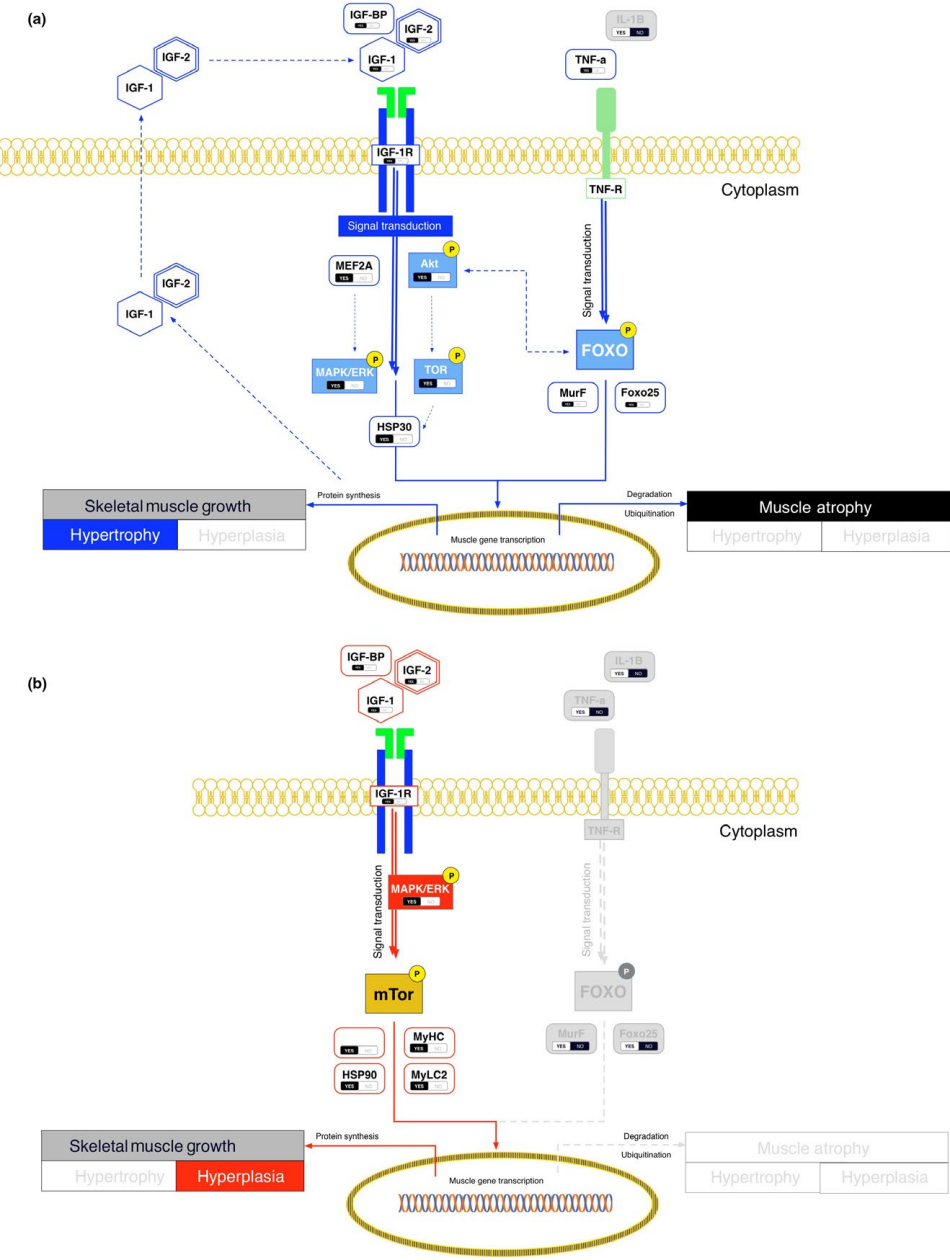
Diagram of muscle‐growth signaling pathway and differentially expressed genes, thus summarizing the effect of thermal gradient on muscle growth. Signaling pathway diagrams for (a) hyperplasic and (b) hypertrophic transcription events in skeletal muscle. IGF complexes mediating interactions with IGF‐IR at the plasma membrane trigger a signaling cascade that links mRNA activation/inhibition through phosphorylation/dephosphorization events

To better understand the effects of temperature range amplitude on growth trajectories, muscle structure, and survival during fish development, the contributions of muscle‐regulatory mRNA components of hypertrophy/hyperplasia were assessed. The temperature ranges significantly impacted myogenesis at different developmental phases. Several mRNAs linked to hyperplasic growth (*myogenic regulatory factors*) increased from the larval to juvenile stages in both thermal ranges (Figures 6 and 7a, b). *Myogenic regulatory factors* such as *murf1* are mechanistically linked to *Myod* and inhibit myogenesis by promoting transcriptional complexes through protein–protein interactions (Olguin, Yang, Tapscott, & Olwin, 2007). Under the RTR, the mRNA levels of *MeF2a, Tnfa,* and *Foxo25* increased in larvae to juveniles (Figure 6), suggesting correspondence between a molecular signature and increased cross‐sectional fiber size or hypertrophy (Bower & Johnston, 2010; Campos et al., 2013; Johnston, Bower, & Macqueen, 2011). Thermal‐induced changes in gene expression have been found in other species. For example, the mRNA abundance of *myog* expression in puffer fish increases at higher temperatures (Fernandes et al., 2006). Furthermore, *Myod1a* and *myog* are tightly linked with an increased muscle fiber quantity (Johnston et al., 2009). Therefore, we postulate that a WTR induces hyperplasic muscle‐growth regulation, which would be indicative of a central role for temperature–behavior integration in controlling systemic regulatory responses.

## 4 DISCUSSION

For most ectothermic species, thermal variations influence growth rates by triggering deep changes to physiological mechanisms, mainly those impacting diffusion rates and enzyme‐substrate complexes (Fry, 1971; Takasuka & Aoki, 2006). For the present study, we hypothesized that coupling of the metabolic/physiological responses to a wide thermoregulatory range would promote survival and increase the performance of biological processes. The mechanisms underlying the impacts of thermal environments (wide vs. restricted thermal ranges) are unknown, although reports do exist for functional advantages in terms of physiology (Killen, 2014) and infection responses (Boltaña et al., 2013). Nevertheless, the present report is the first to provide evidence that the adaptive value of thermal behavior may depend on (1) temperature range amplitude, (2) the ability of the fish to choose a preferred temperature according to needs, and (3) the interaction thereof with metabolic mechanisms that, in turn, influence systemic regulatory systems during development.

In ectotherms, data for temperature influences on growth performance are mainly based on individuals adapted to temperatures kept constant for days or weeks (Albokhadaim et al., 2007; Claireaux & Lagardère, 1999; Elliott & Hurley, 1997; Elliott, Hurley, & Fryer, 1995; Galloway, Kjorsvik, & Kryvi, 1999; Johnston, 2003; Johnston et al., 2009; Johnston, McLay, Abercromby, & Robins, 2000; Macqueen et al., 2008; Martell & Kieffer, 2007; Nathanailides, Stickland, & Lopez‐Albors, 1995). However, details on thermal variations and on subsequent impacts to thermoregulation have received scarce investigative attention. It is commonly recognized that acclimation to different thermal environments affects the physiological response at several regulatory levels. While there is a considerable body of work on the consequences of thermal stress, the majority focuses on how the physiological responses have been influenced by thermal differences in the environment. Thermal stress notably impacts physiological performance, namely in terms of growth trajectories, genetic structure, and/or survival (Bradbury et al., 2010; Houde, 2008; Killen et al., 2010; Meier et al., 2014). Experimental designs using constant temperatures provide insight into the limits of the physiological system over the course of acclimation to extreme thermal conditions; however, a dynamic thermal design with fluctuating temperatures can elucidate optimization mechanisms of the regulatory response, such as in growth, physiology, metabolism, or the defense response.

The presently obtained results suggest that thermal heterogeneity substantially impacts the muscle structure and growth trajectories of *S. salar*. Growth trajectories, including morphometric/histology parameters, muscle microstructure, and gene expression regulation, significantly differed between WTR and RTR individuals. The size trajectory of RTR individuals displayed similar growth patterns to those observed in other fish species conditioned to restricted temperatures (Johnston et al., 2003; Valente et al., 1999). These results are also in accordance with the oxygen supply hypothesis, which suggests that restricted or cold temperatures are related to high energetic costs that modify mitochondrial function, thus finally impacting the size of the individual (Atkinson, Morley, & Hughes, 2006). Additionally, the reduced growth performance registered in RTR individuals suggests that development under a constant temperature may incur high energetic costs associated with restricted thermoregulation during development. In contrast, the energetic demands in WRT are optimized by the ability to freely move across a large temperature range (Chown & Nicolson, 2004), which supports the assumption of temperature dependence in *S. salar* ontogeny (Cavieres, Bogdanovich, & Bozinovic, 2016).

The current results further suggest that a WTR during development acts to fine tune the response of regulatory mechanisms and to promote the improvement of highly specific regulatory traits, whereas a RTR appears to induce decreased growth performance and increased mortality. In ectotherms, the beneficial effects of thermoregulation during pathogenic challenges are established (Cerqueira et al., 2016; Killen, 2014). Studies in zebrafish have also shown that a WTR drives a coherent and strong immune response, with significant effects on adaptive mechanisms such as survival (Boltaña et al., 2013). The ability of ectotherms to increase muscle fiber size (hypertrophy) or quantity (hyperplasia) during development is highly dependent on temperature (Devoto et al., 2006; Johnston, Cole, Abercromby, & Vieira, 1998; Johnston et al., 2003). In particular, the present results suggest that the impact of temperature range on muscle cellularity directly affects final size. A similar pattern has been observed in wild brown trout (*Salmo trutta*), where resident individuals conditioned within restricted thermal environments are smaller in size (Jensen et al., 2008; Meier et al., 2014). The significance of this metabolism–environment interaction was highlighted by SHG analysis, specifically by extending the interpretative value of a one‐dimensional morphological response into regulatory modules where the effect of temperature may operate. RTR fish showed cellular conditions that were more susceptible to muscular microstructure injury, as based on recorded sarcomere lengths.

In fish, differences in muscle structure have been attributed to the chemical degradation of myosin filaments in sarcomeres (Huang et al., 2011). As myosin is an integral component of the sarcomere unit, with a fundamental role in muscle contraction, changes to myosin length can result in reduced muscular function (Mohaupt et al., 2009). Such changes could lead to significant modifications in metabolic mechanisms that, in turn, could negatively impact the resilience of individuals inhabiting environments with thermal constants, such as under aquaculture or laboratory conditions. The results of the present study indicate that optimization of thermal preference through thermoregulation can increase the efficacy of the regulatory metabolic system, particularly in optimizing muscle growth, muscle structure, cellularity, and, ultimately, final size.

The observed results further support prior data on significant differences in muscle‐related mRNA transcripts for specific regulatory components of hyperplasic and hypertrophic growth (Bower & Johnston, 2010; Campos et al., 2013; Johnston et al., 2011). More specifically, thermal range amplitude significantly and indistinctly impacted muscle growth during the various developmental phases of *S. salar*. A significant effect was found for the WTR on regulatory components of hyperplasic growth, where *myod1a* and *myog* mRNAs were upregulated. *Myod1a* and *myog* are temperature‐dependent in other fish species, with increased temperatures linked to increased mRNA abundances (Fernandes et al., 2006; Johnston et al., 2009). In contrast, the abundances of *MRF, MeF2a, Tnfa,* and *Foxo25* mRNAs were increased in RTR individuals. This suggests that a constant temperature may inhibit hyperplasic regulatory mechanisms during early development while escalating hypertrophic regulation to increase fiber sizes, thus creating inhibitive molecular conditions for hyperplasic muscle growth.

In conclusion, the present study employed a novel experimental setup that ultimately revealed the effects of juxtaposed thermal environments on the fine‐tuned mechanisms of fish growth. The obtained results suggest that temperature range amplitude strongly and significantly affects fish development. Therefore, we propose that a wide temperature range successfully promotes diverse biological responses on a limited temporal scale by acting as an integrative signal that orchestrates several biological outputs during development (Jensen et al., 2008; Meier et al., 2014). This, in turn, would increase the efficacy of metabolic machinery and provide a positive adaptive value to individuals. The observed metabolic/molecular thermal synergy, and consequently increased survival, sheds further light on the physiological and metabolic implications of rearing fish at constant temperatures, such as occurs under aquaculture or laboratory settings. Finally, the currently presented results contribute toward comprehending the hypothetical impacts of global warming and the potential effects thereof on thermoregulatory behavior in ectotherms.

## CONFLICT OF INTEREST

The authors declare no competing financial interests.

## AUTHOR CONTRIBUTIONS

SB conceived, designed, and coordinated the study. NS and AA carried out the molecular laboratory work and participated in data analysis. GA and SB carried out statistical analysis. CG, DR, and SB drafted the manuscript. All authors gave final approval for publication.
