# Supplementary figures and images for: Influences of thermal environment on fish growth
Source: Ecol Evol. 2017 Jul 26;7(17):6814–25. doi: 10.1002/ece3.3239 (PMC5587470; doi:10.1002/ece3.3239)

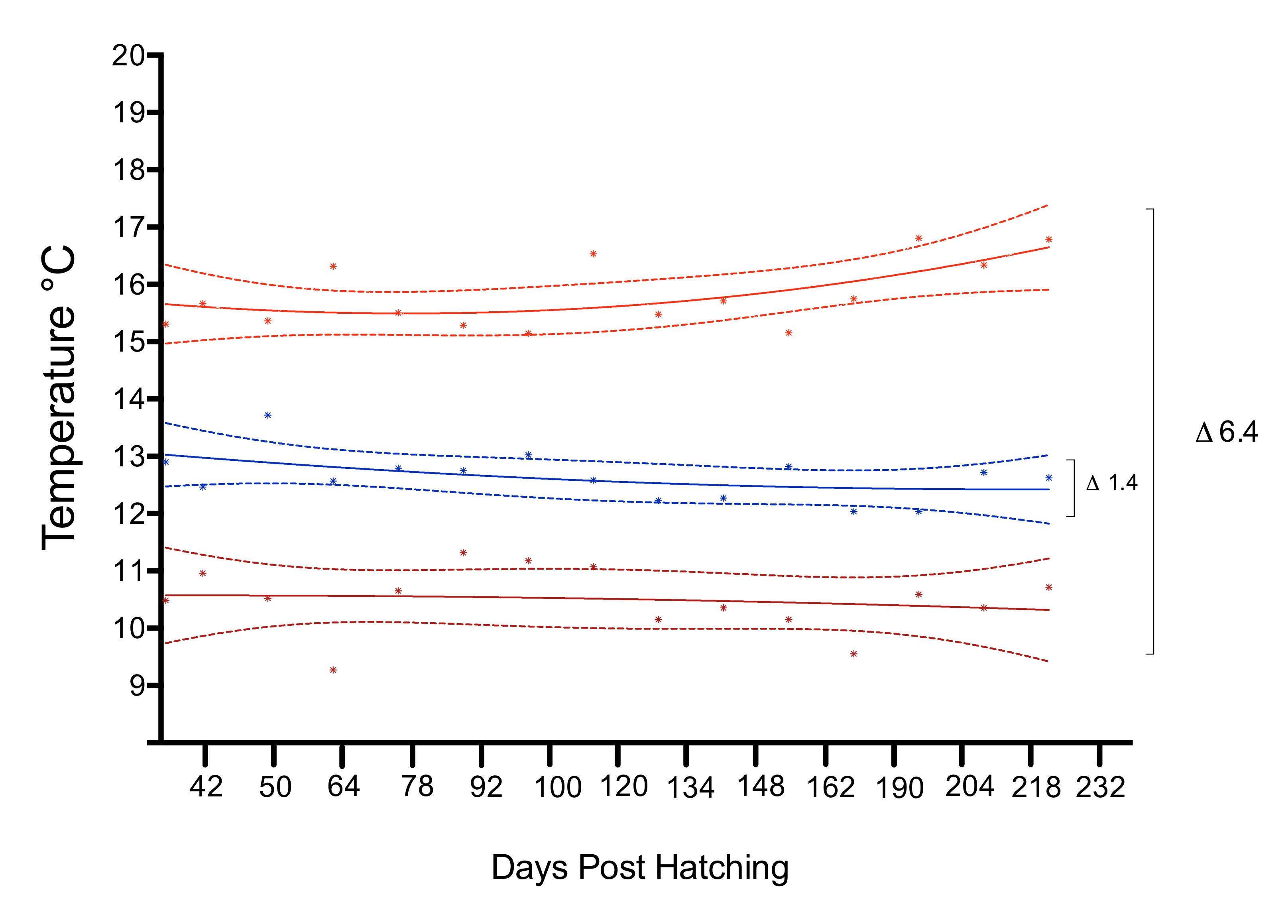

Supplement: Supplementary file 1 [file ECE3-7-6814-s001.jpg]

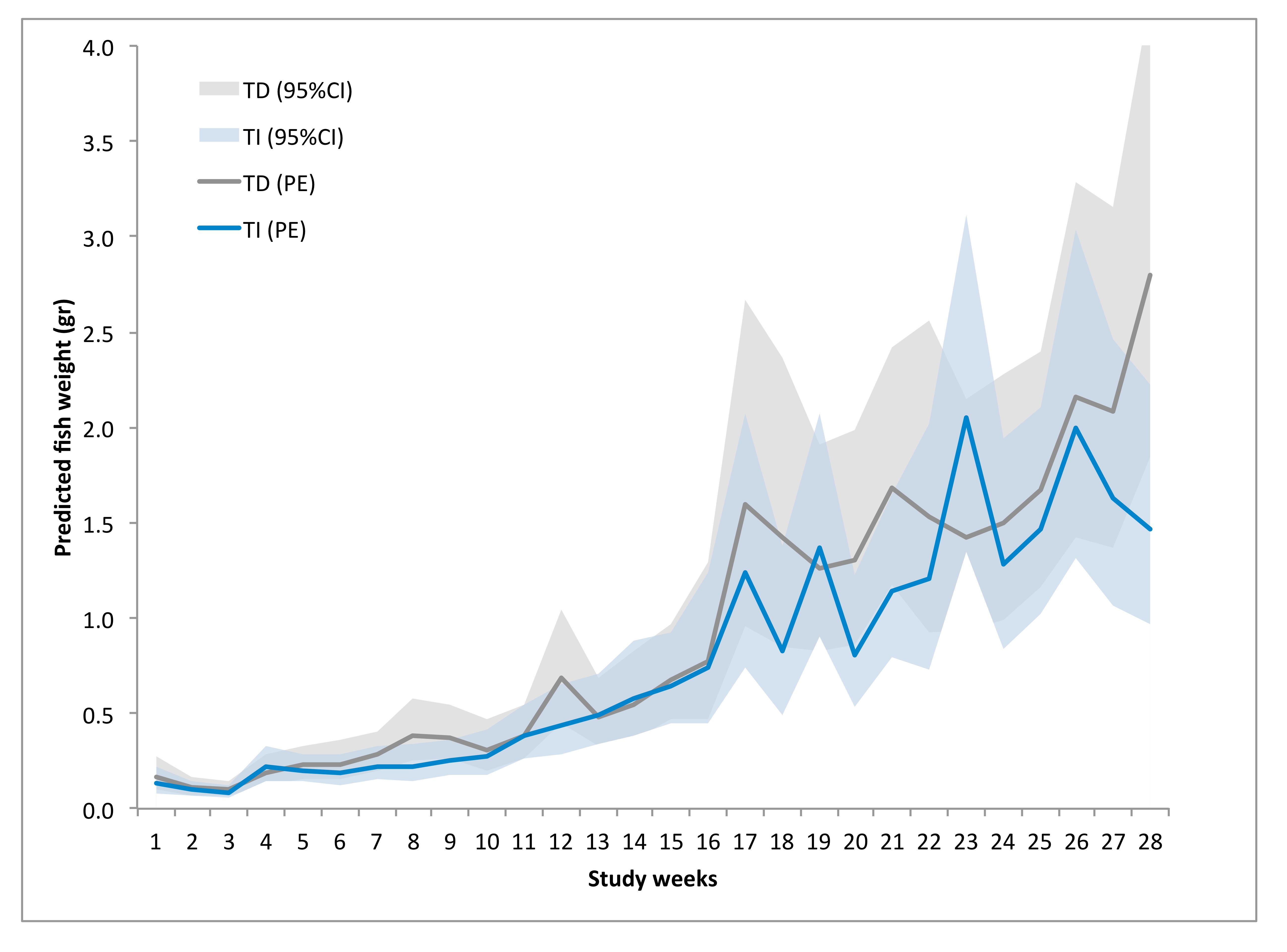

Supplement: Supplementary file 2 [file ECE3-7-6814-s002.jpg]

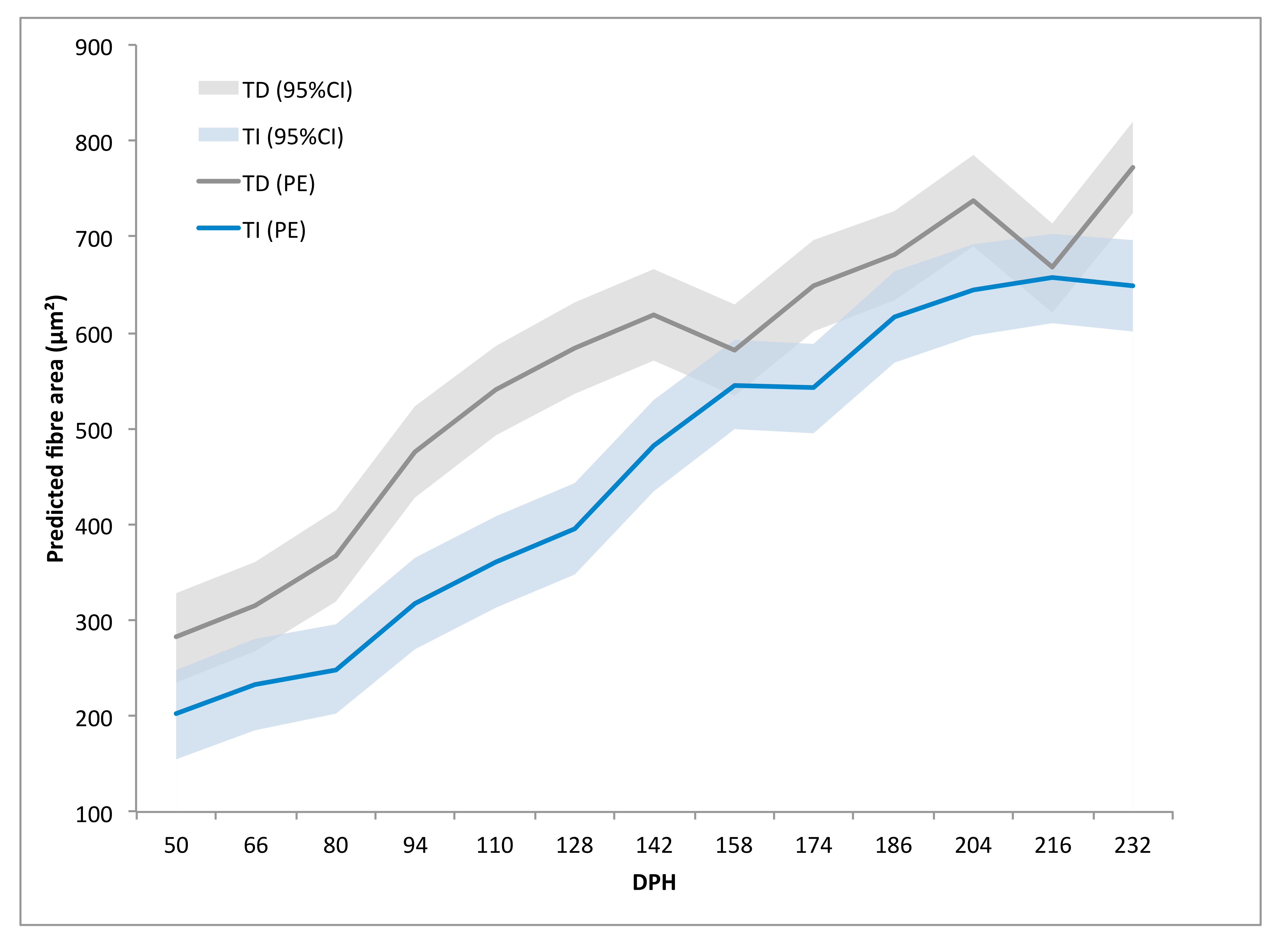

Supplement: Supplementary file 3 [file ECE3-7-6814-s003.jpg]
