# Supplementary material for: Influences of thermal environment on fish growth
Source: Ecol Evol. 2017 Jul 26;7(17):6814–25. doi: 10.1002/ece3.3239 (PMC5587470; doi:10.1002/ece3.3239)
Supplement: Supplementary file 4 [file ECE3-7-6814-s004.docx]

| Gene | Primer 5'-3' | Product size (bp) | E (%) | Access Number | Author |
| --- | --- | --- | --- | --- | --- |
| IGF-I | F: CCTGTTCGCTAAATCTCACTTC | 226 | 102 | EF432852 | Valente et al., (2012) |
|  | R: TACAGCACATCGCACTCTTGA |  |  |  |  |
| IGF-II | F: GGAAAACACAAGAATGAAGGTCAA | 127 | 82 | EF432854 | Valente et al., (2012) |
|  | R: CCACCAGCTCTCCTCCACATA |  |  |  |  |
| IGF-IRa | F: GGGGCTCTCCTTCTGTCCTA | 175 | 97 | EU861008 | Valente et al., (2012) |
|  | R: AGAGATAGACGACGCCTCCTA |  |  |  |  |
| HSP30 | F: CCGTTCAGGCAGATCAAACT | 135 | 94 | NP001134440 | García de la Serrana & Johnston (2013) |
|  | R: GAGGAGCTGTCTGTCAAGCA |  |  |  |  |
| HSP90α1a | F: AAAAAAACAGGAGGAGCTGAATT | 259 | 95 | KC150878 | García de la Serrana & Johnston (2013) |
|  | R: ATGTTGGCTCACCCGTAGTTG |  |  |  |  |
| Miogenina | F: GTGGAGATCCTGAGGAGTGC | 146 | 101 | DQ294029 | Bower et al. (2008) |
|  | R: CTCACTCGACGACGAGACC |  |  |  |  |
| FBXO25 | F: CCTTGGTAACATAATGTATGTCC | 137 | 102 | DN165813 | Bower et al. (2008) |
|  | R: CCAGGAAATGAATGATAATACC |  |  |  |  |
| MEF2A | F: ACCGGCTACAACACCGAGTA | 121 | 102 | DY713536 | Bower et al. (2008) |
|  | R: CCTGGCCCAGTTGATGTT |  |  |  |  |
| MuRF1 | F: AGGCGGGATCAGAGCTAAC | 229 | 100 | DN165465 | Bower et al. (2008) |
|  | R: CGACCATTCCAAAGTCCATC |  |  |  |  |
| MyHC | F: GCACGCCACTGAAAAC | 209 | 95 | DN164736 | García de la Serrana & Johnston (2013) |
|  | R: CCTCAAGGTCGTCCACT |  |  |  |  |
| mlc2 | F: TCAACTTCACCGTCTTCCTCAC | 194 | 98 | NM001123716 | García de la Serrana & Johnston (2013) |
|  | R: GCCCACAGGTTCTTCATCTCC |  |  |  |  |
| fkbp4 | F:ATGAAGGAGCTGCCGTAGAA | 161 | 161 | NM001140424 | García de la Serrana & Johnston (2013) |
|  | R:GAGGGACTCTTCTCCCTGCT |  |  |  |  |
| myf5 | F: CGCCATCCAGTACATCGAG | 213 | 100 | DQ452070 | Valente et al., (2012) |
|  | R: TCTCCAGAGCTCACATTCTTAGTAT |  |  |  |  |
